# Signatures of Drug Sensitivity in Nonsmall Cell Lung Cancer

**DOI:** 10.1155/2011/215496

**Published:** 2011-08-07

**Authors:** Hua C. Gong, Sean Wang, Gary Mayer, Guoan Chen, Glen Leesman, Sharat Singh, David G. Beer

**Affiliations:** ^1^Department of Research and Development, Prometheus Inc. 9410, Carroll Park Drive, San Diego, CA 92121, USA; ^2^Department of Surgery, School of Medicine, University of Michigan, 6304 Cancer Center, Ann Arbor, MI 48109, USA; ^3^Cancer Center, University of Michigan, Room 6304, Ann Arbor, MI 48109, USA

## Abstract

We profiled receptor tyrosine kinase pathway activation and key gene mutations in eight human lung tumor cell lines and 50 human lung tumor tissue samples to define molecular pathways. A panel of eight kinase inhibitors was used to determine whether blocking pathway activation affected the tumor cell growth. The HER1 pathway in HER1 mutant cell lines HCC827 and H1975 were found to be highly activated and sensitive to HER1 inhibition. H1993 is a c-MET amplified cell line showing c-MET and HER1 pathway activation and responsiveness to c-MET inhibitor treatment. IGF-1R pathway activated H358 and A549 cells are sensitive to IGF-1R inhibition. The downstream PI3K inhibitor, BEZ-235, effectively inhibited tumor cell growth in most of the cell lines tested, except the H1993 and H1650 cells, while the MEK inhibitor PD-325901 was effective in blocking the growth of KRAS mutated cell line H1734 but not H358, A549 and H460. Hierarchical clustering of primary tumor samples with the corresponding tumor cell lines based on their pathway signatures revealed similar profiles for HER1, c-MET and IGF-1R pathway activation and predict potential treatment options for the primary tumors based on the tumor cell lines response to the panel of kinase inhibitors.

## 1. Introduction

Lung cancer is the leading cause of cancer-related deaths worldwide, resulting in 1.61 million new cases and 1.38 million deaths per year according to the Global cancer statistics estimation in 2011 [1]. Lung cancer is generally classified histologically into two major types, small cell lung cancer (SCLC) and nonsmall cell lung cancer (NSCLC). Approximately 85–90% of lung cancers are NSCLC representing three major subtypes based on tumor cell size, shape, and composition, with adenocarcinoma accounting for 40%, squamous cell lung carcinoma 25–30%, and large-cell lung carcinoma accounting for 10–15% of all lung cancers [2, 3]. Although less than optimal, current conventional treatment for lung cancer consists of surgery for operable candidates and chemotherapy for disease-advanced patients with the mean survival for most advanced lung cancer patients less than one year [4]. During the last decade, considerable progress has been made in the treatment of NSCLC due to the emergence of new targeted therapies specific to the oncogenic tyrosine kinase pathways activated in tumor cells. For example, two epidermal growth factor receptor (HER1) tyrosine kinase inhibitors (TKI), Gefitinib (Iressa) and Erlotinib (Tarceva), have been FDA approved for the treatment of locally advanced or metastatic NSCLC that has failed at least one prior chemotherapy regimen [5, 6]. Other receptor tyrosine kinase (RTK) pathway inhibitors, such as Sunitinib (Sutent), which targets the platelet-derived growth factor receptors and vascular endothelial growth factor receptors, as well as Crizotinib, a hepatocyte growth factor RTK inhibitor, are in advanced clinical trials for NSCLC [7, 8]. 

The advances made in targeted therapy for NSCLC are based on understanding the mechanism by which mutated genes confer a neoplastic phenotype on tumor cells and how the targeted interruption of these oncogenic pathways leadsto clinical response. Thus, analysis of a pathway-focused panel of biomarkers in fresh tumor tissue samples collected from patients could pave the way for determining if the markers are associated with the optimal clinical therapy and may provide predictive value in identifying responsive patients. In addition, drug combinations targeted against the receptors affecting downstream signaling molecules may overcome pathway activation and drug resistance often seen in NSCLC therapy. Difficulties in predicting efficacy in targeted therapy is due to the limited knowledge of the activated oncogenic pathways in the patient's tumor so that the appropriate inhibitor(s) are not prescribed. Thus, preclinical cellular response profiling of tumor tissue samples has become a cornerstone in the development of novel cancer therapeutics. To this end, we have developed and trademarked a channel enzyme enhanced reaction (CEER) assay methodology to profile some of the major oncogenic pathways activated in cancer cells and have used this assay together with genotyping to characterize the activated oncogenic pathways in eight human NSCLC tumor cell lines as well as 50 fresh-frozen NSCLC samples collected from patients. The aim of this study was to assess the potential to prospectively classify lung cancer patients into different treatment groups based on correlation of pathway activation profiles, gene mutational status, and clinical features between the patient tumor samples and the tumor cell lines. In addition, we evaluated the efficacy of a panel of eight kinase pathway inhibitors to block the pathway activation and proliferation of these eight lung tumor cell lines and used the results to identify treatment options for the 50 lung cancer patients.

## 2. Materials and Methods

### 2.1. Human Lung Tumor Cell Lines, Lung Cancer Tissue Samples, and Kinase Inhibitors

Eight NSCLC cell lines, HCC827, H1975, H1734, H1993, H358, H1650, A549, and H460, were selected, and they represent the major NSCLC cancer subtypes, adenocarcinoma and large-cell lung carcinoma. The cell lines were purchased from ATCC (Table 1). Fifty lung adenocarcinomas samples were collected from patients operated on for lung cancer at the University of Michigan. Collection and use of all tissue samples were approved by the Human Subjects Institutional Review Boards of the University of Michigan. The demographic information of the patients is shown in Supplementary Table 1. The primary tumor samples were snap frozen and cryostat-sectioned to identify regions representing >70% tumor cellularity for subsequent pathway analysis. The samples (~2 cubic millimeters in size) were shipped to Prometheus Laboratories on dry ice for analysis. Eight kinase inhibitors representing a diverse panel of potential cancer therapeutics were purchased from Selleck Chemicals (Houston, TX). The collection included specific as well as multiple RTK inhibitors, that is, compounds targeting the cellular kinase pathways: HER1/2/4 (epidermal growth factor receptors) inhibitors (Erlotinib for HER1, Lapatinib for HER1/2, Gefitinib for HER1/2/4, and BIBW-2992 is an irreversible inhibitor for HER1/2); c-MET (hepatocyte growth factor receptor) inhibitor, PF-2341066; IGF-1R (insulin-like growth factor-1 receptor) inhibitor BMS-536924; MEK (mitogen-activated protein kinase kinase) inhibitor, PD-325901, and PI3K (phosphatidylinositol-3-kinase) and mTOR (mammalian target of rapamycin) inhibitor BEZ-235.

### 2.2. Preparation of Lysates from Cell Lines and Primary Tumor Samples

Tumor cells were cultured in their respective growth medium recommended by ATCC plus 10% fetal bovine serum (FBS). Cells were grown in 35 mm 6-well cell culture plates until reaching 80% confluence. After washing the cells with phosphate buffered saline (PBS) 3 times, the cell culture plate was placed on ice and then the plate was carefully tilted on its side for 10 sec to completely remove all residual media. Then, 150 *μ*L of ice cold lysis buffer was added to each plate and the plate was then left on ice for 5 min. The lysed cells were scraped off and together with the crude lysate transferred to a 1.5 mL centrifuge tube. The mixture was vortexed in the tube, placed on ice for 15 min and then centrifuged at 14,000 rpm for 15 min at 4°C. The supernatant was transferred to another centrifuge tube and stored at −70°C until analysis. The frozen tumor samples were similarly processed by the addition of 4 volumes of ice-cold lysis buffer per tissue volume and homogenized in a Powergren High Throughput Homogenizer (Fisher Scientific) at a speed setting of 7 for 2 min. The homogenate was transferred to a 1.5 mL centrifuge tube and centrifuged at 14,000 rpm for 15 min, at 4°C. The supernatant from the tumor lysate was harvested and stored at −70°C until analysis.

### 2.3. Profiling of Signaling Pathways Using the CEER Assay in Tumor Cell Lines and Tissue Samples

The principle of the CEER assay is based on the capture of the target protein by a target-specific antibody printed in two dilutions on the surface of a microarray slide. Measurement of the activation status of the captured target protein is revealed by the formation of a unique immunocomplex, requiring the colocalization of two detecting enzyme-conjugated antibodies on the same target protein captured on the microarray surface as illustrated in Supplemental Figure 1. Formation of this complex is initiated by the binding of the first detecting antibody, which is coupled to glucose oxidase (GO), to an epitope on the captured target protein that is different from the epitope recognized by the capture antibody, followed by the binding of a second detecting antibody, which is coupled to horseradish peroxidase (HRP), to a phosphorylated tyrosine (p-Tyr) residue on the target protein. Upon the addition of glucose, the immobilized GO on the captured target protein produces H_2_O_2_ and due to the close proximity, the locally generated H_2_O_2_ is then utilized by the HRP coupled to the p-Tyr-specific second detecting antibody to generate a chemical signal that can be amplified with biotinyl-tyramide. The sensitivity and specificity for the detection of the phosphorylated target protein are greatly enhanced by this collaborative reaction and amplification process, which is mediated by the simultaneous binding of three different antibodies on the same target protein [9] (for details of the CEER assay method to profile the tumor cell lines and tissue samples see Supplemental Methods).

### 2.4. Inhibition of Activated Signaling Pathways in Tumor Cell Lines by Kinase Inhibitors

The tumor cells were cultured in their respective growth medium with 10% FBS in 35 mm 6-well cell culture plates until they reached ~80% confluence. The cells were then starved overnight in serum-free medium, followed by a 4-hour treatment with various concentrations of the kinase inhibitor. Afterwards, cell lysates were prepared from the treated cells as before and aliquots of the lysates subjected to the CEER assay.

### 2.5. Inhibition of Tumor Cell Line Growth by Kinase Inhibitors

The tumor cells were seeded into 96-well cell culture plates and maintained in culture for 24 hours. After washing, the cultured cells were incubated in their respective medium containing 5% FBS and various concentrations of the indicated inhibitor for 48 hr. Determination of tumor cell growth inhibition was performed by adding 100 *μ*L of the combined Cell Titer-Glo Buffer and Cell Titer-Glo Substrate Labeling Reagent (Promega) to each well of the plates, followed by incubation at room temperature for 10 min to stabilize the luminescence. The luminescent signal from the cell samples was detected by using an M5 microtiter plate reader. For studies involving treatment with more than one inhibitor, the selected inhibitor that showed more than 25% inhibition of tumor cell growth at 10 *μ*M concentration when treated individually was further tested in combination treatment with another inhibitor. A 5 *μ*M concentration of each inhibitor was combined to make a 10 *μ*M dose, and the same half log dilution was made as in the single drug treatment for adding to the cells. Tumor cells were treated for 48 hr and cell viability was measured as in the single inhibitor treatment.

### 2.6. Anchorage-Independent Inhibition of Tumor Cell Line Growth by Kinase Inhibitors

A single cell suspension of 3000 cells from each of the eight tumor cell lines in 1 mL mixture of 1.2% Agarose (Seaplaque; FMC, Rockland, ME) in DMEM (Life Technologies, Carlsbad, CA) plus 10% FBS was added on top of 1% soft agarose that had been allowed to gel previously in the wells of a 35 mm 6-well cell culture plate. The plates were kept at 4°C for 2 hr to solidify the cell-containing layer. The plates were then incubated at 37°C in a CO_2_ incubator with 2 mL of medium containing various concentrations of the kinase inhibitor. The medium with the inhibitor was changed every 3 days. After 2 weeks, cell colonies larger than 10 cells were scored under a Nikon inverted-phase microscope [10].

### 2.7. Genotyping of Lung Tumor Tissue Samples

Genomic DNA was isolated from human tumor tissue samples using the DNeasy kit (Qiagen, Valencia, CA). Primers and probes for all of the measured SNPs were obtained from the ABI TaqMan SNP Genotyping Assay (Applied Biosystems), using the Assay-by-Design service for which we provided the sequences, or the Assay-on-Demand service when the assays were already designed by Applied Biosystems. Reactions were performed in 5 *μ*L volume and contained 10 ng DNA, 1x TaqMan Universal Mastermix (Applied Biosystems), 200 nM of each probe and 900 nM of each primer. Cycling conditions on the ABI PRISM 7900HT Sequence Detection System (Applied Biosystems) were 10 min at 95°C, followed by 40 cycles of 15 sec at 95°C and 1 min at 60°C. After cycling, the endpoint fluorescence was measured and the amplified sequences determined by DNA sequencing analysis. Alleles were assigned using the SDS 2.1 software (Applied Biosystems).

### 2.8. Lung Tumor Tissue Sample Clustering Analysis and Heat Map Generation

Hierarchical clustering analysis was performed on the 50 lung tumor tissue samples to explore whether the pathway activation profiles determined by the CEER assay and the gene mutational analysis done for these samples could segregate them into distinct subsets that are similar to the pathway activation and mutational signatures of the tumor cell lines. The general construction of a hierarchical agglomerative classification was achieved by using an algorithm to find the two closest objects and merge them into a cluster, and then find and merge the next two closest points, where a point is either an individual object or a cluster of objects. A heat map of one-dimensional hierarchical clustering result was generated in the analysis to demonstrate the sample clustering structure based on pathway activation signatures and mutational status.

## 3. Results

### 3.1. Profiling of the Activated Kinase Pathways in Tumor Cell Lines

**Figure 1:**
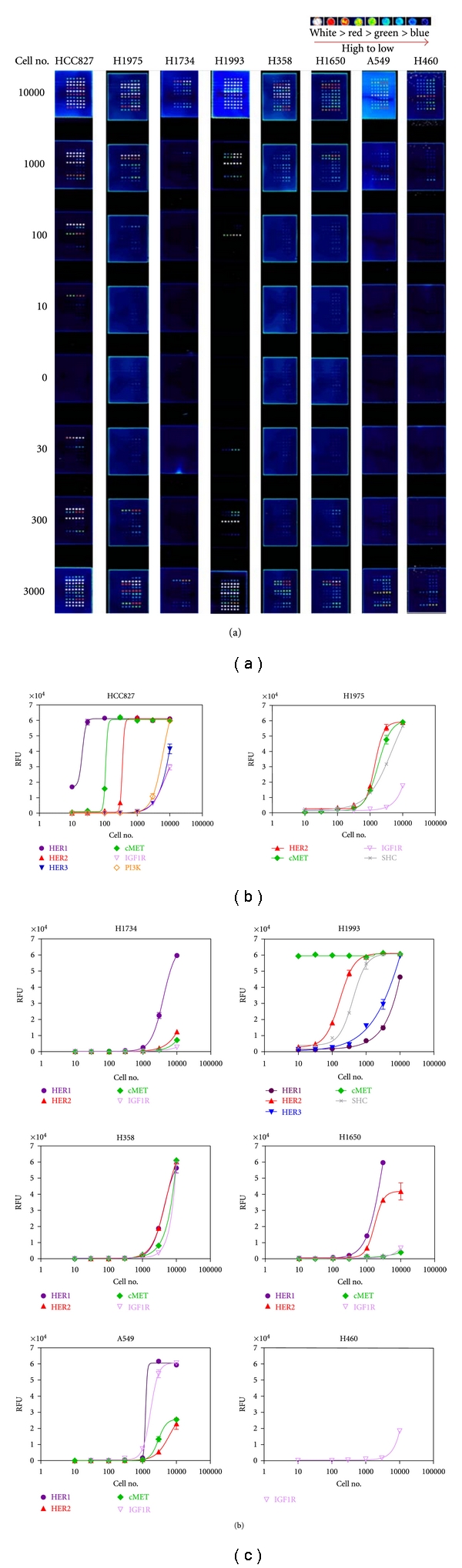
Profiling of the activated (p-Tyr) RTK signaling pathways in the lung tumor cell lines. (a) Slide images of the CEER assay obtained from the eight tumor cell lines (please see the supplemental data and methods for experimental details available online at doi: 10.1155/2011/215496). (b) Graphical representation of the activated (p-Tyr) RTK pathways in the lung tumor cell lines profiled by the CEER assay. The cells were cultured in the presence of 10% FBS in their respective medium until 80% confluence and cell lysates were prepared in lysis buffer. The activation signal was determined from the harvested lysate. In each cell line the activated pathways are shown in RFU (relative fluorescent units) based on the lysate obtained from the number of cells being assayed.

The activated cell signaling pathways for eight lung tumor cell lines were profiled using the CEER assay. The assay measured the activated (p-Tyr) HER1, HER2, HER3, c-MET, IGF-1R, c-Kit, PI3K, and SHC levels in the cells based on the number of cells being assayed. The tumor cells were cultured in presence of 10% FBS and harvested at about 80% confluence for preparation of cell lysates and signaling pathway profiling. Serial dilutions of tumor cell lysates equivalent to 10–10,000 cells were assayed, and the raw data captured on the slides are shown in Figure 1(a) and a graphic representative of the data is shown in Figure 1(b). As seen in Figure 1(b), the dose-response curves representing the level of activation in each RTK pathway is inversely proportional to the number of cells being assayed. The more activated the pathway, the less number of cells are needed to generate the maximal signal. Thus, the relative activation of the activated pathways can be determined based on the EC_50_ value of the number of cells being assayed in each pathway activation curve (see Supplemental Table 2). 

As shown in Figure 1(b), each of the eight tumor cell lines exhibited a distinct RTK activation pattern. The lung adenocarcinoma cell line, HCC827, exhibited the greatest number of activated RTK pathways, with HER1, c-MET, and HER2 being highly activated, PI3K being moderately activated, and HER3 as well as IGF-1R being lowly activated. The other adenocarcinoma cell line H1975 showed only moderate activation of HER2, c-MET, and SHC pathways and a low activation of the IGF-1R pathway. The remaining adenocarcinoma cell line H1734 exhibited a moderate activation of the HER1 pathway and a very low activation of the HER2 and c-MET pathways. By contrast, the adenocarcinoma cell line H1993 from a metastatic tumor showed a very potent activation of the c-MET pathway, with also a high activation of the HER2 and SHC pathways, and a moderate activation of the HER3 and HER1 pathways. Both primary and metastatic bronchioalveolar carcinoma cell lines H358 and H1650 showed a moderate activation of the HER1 and HER2 pathways, with H358 also exhibited a moderate activation of the c-MET and IGF-1R pathways. The large-cell carcinoma cell line A549 exhibited a moderate activation of the HER1 and IGF-1R pathways with a low activation of the c-MET and HER2 pathways, whereas the metastatic large-cell carcinoma cell line H460 showed only a very low activation of the IGF-1R pathway.

### 3.2. Inhibition of Activated Signaling Pathways in Tumor Cell Lines by Kinase Inhibitors

**Figure 2:**
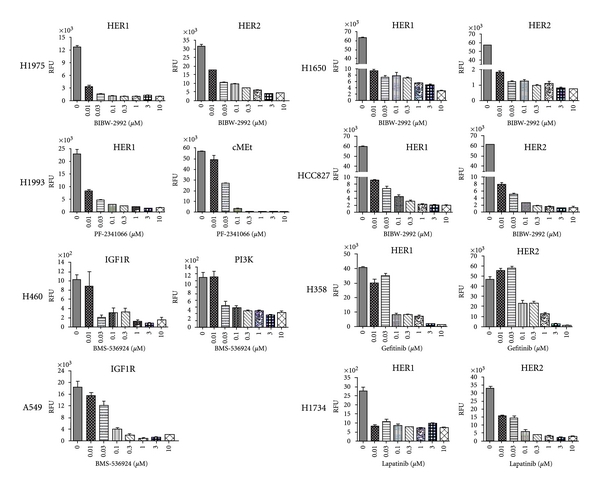
Inhibition of signaling pathway activation in lung tumor cell lines by kinase inhibitors. Lung tumor cells were cultured in 10% FBS until reaching ~80% confluence and then the cells were starved in serum-free medium for overnight, followed by 4-hour treatment with the inhibitors. Cell lysates were then prepared and used for determination of the pathway activation signals by the CEER assay.

Profiling of the eight tumor cell lines showed that the HER1 and HER2 pathways are highly activated in the HCC827 cells and thus treatment of these cells with an irreversible HER1/2 inhibitor BIBW-2992 should be able to block the activation of these pathways. Indeed, the data presented in Figure 2, showed that a potent dose-dependent inhibition of the HER1 and HER2 pathways in the HCC827 cells was observed by treatment with BIBW-2992. Other HER1 and/or HER2 pathway-activated cell lines, H1975 and H1650, were likewise had these pathway activations blocked by the treatment with BIBW-2992. What is remarkable is that the H1975 cell line harbors the T790M and L858R mutations in the HER1 gene (information obtained from the Sanger Institute website), which confer resistance to HER1 kinase inhibitors, Gefitinib and Erlotinib, responded to the irreversible HER1 kinase inhibitor BIBW-2992. Two other additional HER1 and/or HER2 pathway-activated cell lines, H358 and H1734, also had their activation blocked by the HER1/2 kinase inhibitors, Gefitinib and Lapatinib, respectively. In the c-MET amplified cell line H1993, activation of this pathway was blocked by the treatment with PF-2341066, a c-MET kinase inhibitor. The c-MET inhibitor also inhibited the HER1 signaling pathway in this cell line, most likely due to crosstalk between the HER1 and c-MET pathways. Treatment with the IGF-1R kinase inhibitor BMS-536924 was able to block the activated IGF-1R pathway exhibited by the A549 and H460 cell lines.

### 3.3. Inhibition of Tumor Cell Line Proliferation by Kinase Inhibitors

**Figure 3:**
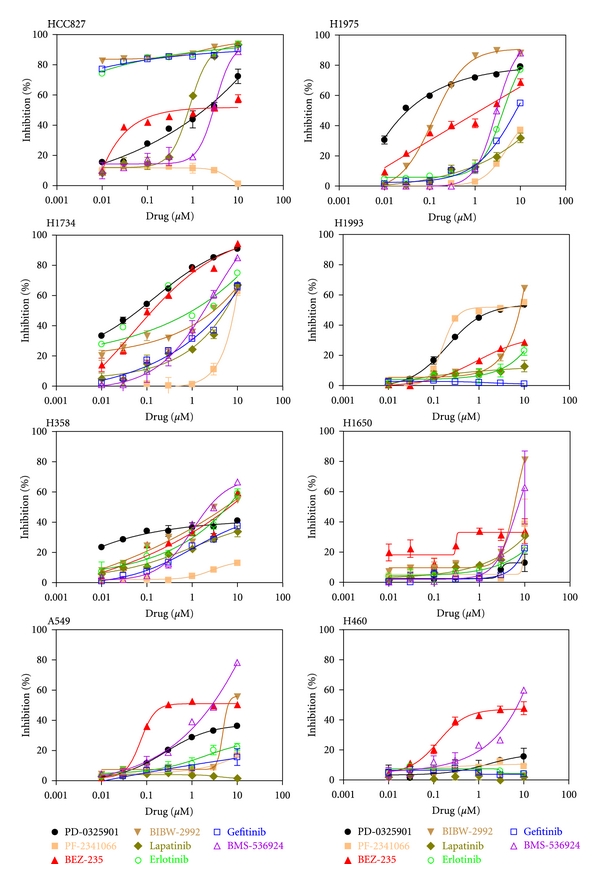
Inhibition of lung tumor cell growth by kinase inhibitors. Lung tumor cells were cultured in 5% FBS plus increasing concentrations of the indicated inhibitors, ranging from 0.01–10 *μ*M, for 48 hours. Determination of cell proliferation was performed with the CellTiter-Glo Luminescent Cell Viability Assay.

Since one purpose of this study was to correlate the activated RTK pathways and gene mutations found in the tumor cell lines with the appropriate kinase inhibitors to determine whether treatment with the inhibitors could inhibit the growth of these cells, the eight tumor cell lines were treated with the selected kinase inhibitors and the results shown in Figure 3. As expected, the lung adenocarcinoma cell line HCC827, which exhibited highly activated HER1 and HER2 pathways as well as HER1 mutation, responded exceedingly well to the HER1 inhibitors, Erlotinib and Gefitinib. In addition, two other HER1/2 inhibitors, Lapatinib and BIBW-2992, were able to inhibit the proliferation of this cell line. By contrast, the specific c-MET inhibitor PF-2341066 (Crizotinib) was not able to inhibit the proliferation of this cell line even though it exhibited the activated c-MET pathway. This is not surprising because it has been shown that Crizotinib is effective in treating patients carrying the ALK/EML4 fusion gene and this drug response is not correlated with c-MET amplification. Moreover, inhibition of proliferation by BMS-536924, an IGF-1R inhibitor, in the HCC827 cells could be due to crosstalk between c-MET and IGF-1R pathways. The proliferation of two other HER1 and HER2 pathway-activated adenocarcinoma cell lines, H1734 and H1975, was also inhibited by Erlotinib. Moreover, the irreversible HER1/2 kinase inhibitor, BIBW-2992, was also able to potently inhibit the growth of the H1975 cell line, which harbored the T790M and L858R mutations in the HER1 gene and thus rendering the cells resistant to Gefitinib and Lapatinib treatment. In addition, the IGF-1R inhibitor BMS-536924 was also able to inhibit the growth of this cell line. In regard to the metastatic adenocarcinoma cell line H1993, which exhibited a potently activated c-MET pathway, the c-MET inhibitor PF-2341066 was able to block its proliferation very effectively. What is more, the downstream MEK inhibitor PD-325901 was also able to block the proliferation of the H1993 cells whereas the irreversible HER1/2 inhibitor BIBW-2992 and the PI3K inhibitor BEZ-235 were able to block the proliferation of this cell line but only weakly. Proliferation of the carcinoma cell line H358, which exhibited a moderate activation of the HER1, HER2, and IGF-1R pathways, was weakly blocked by the HER1/2 inhibitors, Lapatinib and Gefitinib, but the IGF-1R inhibitor BMS-536924 was able to inhibit its proliferation more effectively. Surprisingly, growth of the H358 cell line was not inhibited by the c-MET inhibitor PF-2341066, even though it exhibited a moderately activated c-MET pathway. Growth of the metastatic carcinoma cell line H1650, which harbored moderately activated HER1 and HER2 pathways, was inhibited by the irreversible HER1/2 inhibitor BIBW-2292. Proliferation of the large-cell carcinoma cell line A549, which exhibited moderately activated HER1 and IGF-1R pathways, was inhibited by the PI3K inhibitor BEZ-235 and by the IGF-1R inhibitor BMS-536924 and weakly by the MEK inhibitor PD-325901. Growth of the remaining metastatic large-cell carcinoma cell line H460, which harbored the PIK3CA gene mutation and an activated IGF-1R pathway, was inhibited by the PI3K inhibitor BEZ-235 but weakly inhibited by the IGF-1R inhibitor BMS-536924.

### 3.4. Anchorage-Independent Growth Inhibition of Tumor Cell Lines by Kinase Inhibitors

**Figure 4:**
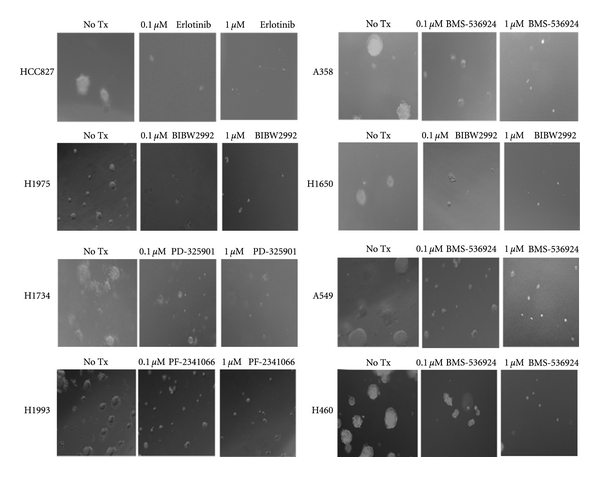
Inhibition of anchorage-independent growth of lung tumor cell lines by selected inhibitors. Each selected cell line was treated with the indicated inhibitor at 0.1 *μ*M and 1 *μ*M concentrations for two weeks and cell colony size formation was scored under the Nikon inverted-phase microscope.

Since anchorage-independent growth is a hallmark of transformed cells, we wanted to ensure that the kinase inhibitors which were able to strongly block tumor cell proliferation in tissue culture were also able to inhibit the same tumor cells' proliferation in an anchorage-independent manner. As seen in Figure 4, Erlotinib, which inhibited the growth of HCC827 cells cultured in anchorage-dependent cell culture plates, was also able to reduce the size of the cell colonies grown in agar plates in an anchorage-independent fashion. Similarly, reduction of cell colony size formation was also observed by the treatment with the irreversible HER1/2 inhibitor BIBW-2992 in H1975 and H1650 cells as seen when these cells were grown in an anchorage-dependent manner. Large colony formation of H1993 cells, whose proliferation in tissue culture was potently blocked by treatment with the c-MET kinase inhibitor PF-2341066, was also inhibited by treatment with the same inhibitor. Proliferation of cell colony size in the H1734 cells was potently blocked by treatment with the downstream MEK inhibitor PD-325901 as seen when these cells were grown in cell culture. Likewise, the IGF-1R kinase inhibitor BMS-536924 was able to reduced cell colony size formation, dose dependently in the H358, A549 and H460 cells.

### 3.5. Inhibition of Tumor Cell Line Growth by a Combination of Two Kinase Inhibitors

**Figure 5:**
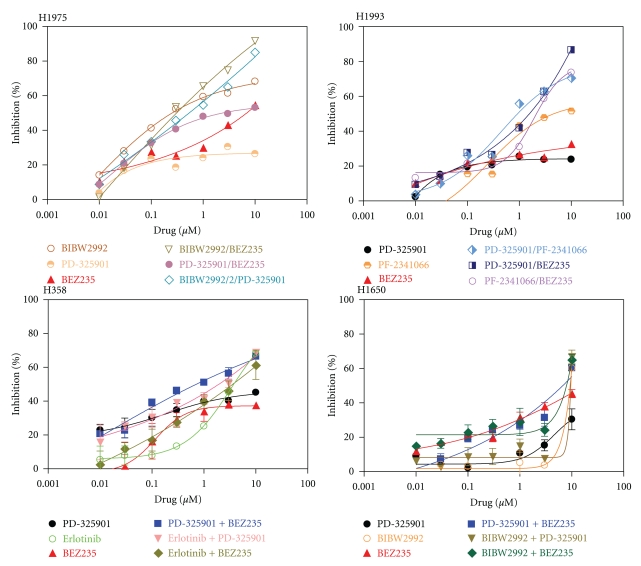
Inhibition of lung tumor cell growth by a combination of two kinase inhibitors. Lung tumor cell lines were cultured in 5% FBS plus increasing concentrations of the indicated single kinase inhibitor or a combination of the two indicated kinase inhibitors. Determination of cell proliferation was performed with the CellTiter-Glo Luminescent Cell Viability Assay.

Tumor cells often rely on signaling from multiple pathways and, hence, treating patients with a single agent can seldom eradicate tumor growth [11]. A common clinical practice is to treat patients with combination therapies. In order to identify therapeutics with synergistic effects, we selected four tumor cell lines out of the eight and treated them with a combination of two kinase inhibitors, one inhibiting the appropriate RTK and the other inhibiting a downstream signaling pathway. As seen in Figure 5, the H1975 cells, whose growth was moderately inhibited by the HER1/2 RTK inhibitor BIBW-2992, but only weakly inhibited by the downstream MEK inhibitor PD-325901 and the downstream PI3K inhibitor BEZ-235, responded more effectively to a combination of BIBW-2992 with either PD-325901 or BEZ-235 with almost 100% growth inhibition of this cell line at 10 *μ*M concentration whereas a combination of the two downstream inhibitors, PD-325901 and BEZ-235, was much less effective. Likewise, combination of PD-325901 or BEZ-235 with the c-MET inhibitor PF-2341066 was more effective in blocking the proliferation of the H1993 cells, which exhibited a potently activated c-MET pathway, then by treating this cell line with the c-MET inhibitor PF-2341066 alone. Interestingly, a combination of both downstream kinase inhibitors, PD-325901 and BEZ-235, was the most effective in blocking the proliferation of this cell line. In the bronchioalveolar cell line H358, which exhibited HER1 and HER2 activation, treatment with the HER1 inhibitor Erlotinib in combination with either one of the two downstream inhibitors, PD-325901 or BEZ-235, showed synergistic inhibition of cell proliferation. A combination of the two downstream inhibitors, PD-0325901 and BEZ-235, was also highly effective. The same phenomenon was observed in the metastatic bronchioalveolar cell line H1650 when it was treated with the irreversible HER1/2 inhibitor BIBW-2992 in combination with one of the downstream inhibitor BEZ-235 or a combination of the two downstream inhibitors, PD-325901 and BEZ-235.

### 3.6. Clustering of Tumor Tissue Samples with Tumor Cell Lines

**Figure 6:**
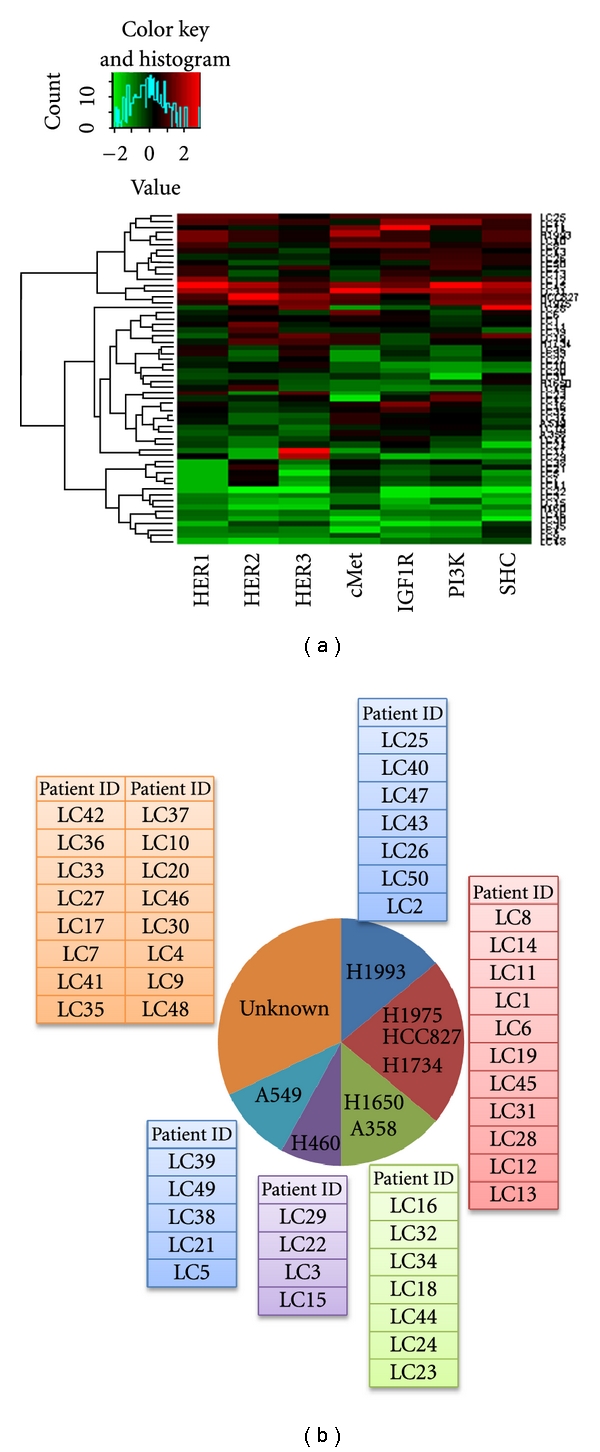
(a) Heat Map representing the activated signaling pathways found in the 50 lung tumor tissue samples and eight lung tumor cell lines. Each row constitutes all the pathway markers determined from an individual tumor sample organized in color columns. Green and red denote markers that are present at lower and higher levels, respectively. (b) Clustering of the 50 lung tumor samples with the corresponding lung tumor cell lines based on the similarities in the markers between the tissue samples and the cell lines.

Profiling of the 50 human lung tissue samples using the CEER assay revealed distinct activated signaling pathways in each of the tissue samples as shown in the heat map in Figure 6(a) (see also Supplemental Table 3). Based on these results, unsupervised one-dimensional clustering of the 50 lung tumor tissue samples with the corresponding cell lines H1993, H1975, HCC827, H1734, H1650, H358, H460, and A549, could be performed as shown in Figure 6(b) because a significant degree of shared pathway activation exists between the primary tumor tissue samples and the tumor cell lines. Clustering of the tumor tissue samples with their corresponding cell lines provides potential insight into targeted therapy based on the drug treatment results obtained from the tumor cell lines.

### 3.7. Gene Mutation Analysis of Tumor Tissue Samples

Mutation analysis of three frequently mutated genes (KRAS, P53, and STK11) in 50 human lung tissue samples further supported the clustering of human tumor samples into the cell line groups (see Supplemental Table 3). For example, patient samples LC16, LC44, and LC23 were originally aligned with tumor cell line H358 based on CEER assay profiling. This alignment was supported by the finding that these three tissue samples also harbored the G34A and G37T KRAS mutations as was found in the H358 cell line. Similarly, the original alignment of patient samples LC45 with cell line H1734 and LC21 with cell line A549 was substantiated by finding the same G34A and G37T KRAS mutations in both patient samples and cell lines. In addition, alignment of the patient sample LC15 with cell line H460 is supported by the finding of the A183T KRAS mutation in both patient sample and cell line. Likewise, the alignment of patient samples LC6, LC31, and LC13 with cell lines H1975 and H1734 was substantiated by the finding of the C726G and G818T P53, mutations in both patient samples and cell lines. Also, the alignment of patient samples LC32 and LC23 with cell line H1650 was supported by the finding of the same P53 mutations in both patient samples and cell line. Lastly, the alignment of patient sample LC25 with the H1993 cell line was supported by the finding of the C109T and G595T STK11 mutations in both patient sample and cell line. Thus, clustering of the patient tissue samples with the tumor cell lines based on mutational status of the KRAS, P53 and STK11 genes was also consistent with the clustering based on the RTK pathway signatures. 

## 4. Discussion

Current lung cancer treatments are less than optimal, with a mean survival of less than one year for advanced lung cancer patients, regardless of treatment regimen [2]. Emerging new treatment modalities are generally targeted to inhibit specific tyrosine kinases activated in the tumor cells through basically two independent approaches [3]. The first approach is to use a highly specific monoclonal antibody to target the membrane growth factor receptor kinase that is responsible for tumor cell growth, and the resulting antibody/antigen complex invokes the host immune system to kill the tumor cells. This approach is exemplified by the treatment of HER2 receptor-positive breast cancer with Herceptin, a humanized monoclonal antibody against this receptor [4]. However, the high cost of monoclonal antibody drugs could be a disadvantage for this approach. The second approach is to develop cell-penetrating small organic molecules that target the specific tyrosine kinases in the signaling pathways of the tumor cells. This approach is best exemplified by the use of Gleevec to block the activation of the BCR-ABL fusion kinase in chronic myelogenous leukemia [5]. The design and synthesis of small molecule tyrosine kinase inhibitors have been greatly facilitated by the availability of crystal structures for the tyrosine kinases in the past decade and effective kinase inhibitors have thus been produced by many large and small pharmaceutical companies. Nevertheless, without prior knowledge of the activated kinase signaling pathways responsible for propagating and metastasis of the tumor cells, it is not possible to apply the targeted therapy approach with the available kinase inhibitors. Therefore, we have selected a panel of eight lung tumor cell lines that harbored the most frequently detected gene mutations: P53, KRAS, STK11, and HER1 as representative examples that cover the major human lung cancer subtypes. 

Mutation analysis has provided valuable information in guiding targeted therapy for cancer patients. A good example is found in cancer patients who carry a KRAS gene mutation because these patients have been shown to be nonresponsive to anti-HER1 therapeutics. Therefore, KRAS mutation testing is becoming routinely performed in patients who are being considered for anti-HER1 therapy with either Cetuximab or Panitumumab in Europe and the United States [12, 13]. Our current study further confirmed an important role of gene mutation analysis in guiding the use of drugs to treat lung cancers. For example, it has been reported that cancers from patients with lung adenocarcinoma that harbored mutations within the tyrosine kinase domain of the HER1 gene often responded initially to TKI drugs such as Gefitinib and Erlotinib [14, 15] but usually developed drug-resistance later [16–18]. Indeed, the HCC827, H1975, and H1650 lung tumor cell lines employed in this study harbored the HER1 gene mutation and they were found to be sensitive to HER1 inhibitor treatment. By contrast, KRAS gene mutation is associated with resistance to HER1 tyrosine kinase inhibitors [19, 20]. This phenomenon is substantiated by our finding that the A549 and H460 cells, which harbored the KRAS mutated gene, did not respond to treatment with HER1 inhibitors but they are sensitive to the downstream PI3K inhibitor BEZ-235. 

Although targeted therapy based on association of somatic mutation analysis and drug sensitivity has greatly facilitated lung cancer treatment, the profiling of oncogenic signal transduction pathways in human tumor cells and tissues offers yet another complementary approach to guide therapeutic treatment [21–25]. Traditional signaling pathway profiling in tumor tissue samples by immunohistochemistry (IHC) staining or Western blotting methods are neither quantitative nor sensitive enough to have utility when only small amount of tumor tissue samples is available. The CEER assay is an assay that we have developed to overcome the low sensitivity and specificity issues associated with these traditional methods. The CEER assay uses a multiplexed, proximity-based, collaborative immunoassay platform that can provide clinical information on a limited amount of tissue samples with high sensitivity and specificity. The principle of the assay is based on the formation of a unique immunocomplex that requires the colocalization of two detecting antibodies against a target protein once the protein is captured on the microarray surface. It is the formation of this complex that enables the generation of a highly specific and sensitive signal to reveal the activation status of the target protein. We have compared CEER with the conventional IHC/FISH and Western blotting and found that the CEER assay provided more quantitative information in regard to the oncogenic kinases [26]. Using this assay, the activated HER1, HER2, HER3, c-MET, IGF-1R, PI3K, and SHC pathways present in the eight lung tumor cell lines as well as the 50 human lung tumor tissue samples were profiled. Cell lines that exhibited one or more of these activated pathways were treated with the corresponding kinase inhibitors and the results demonstrated that this matching approach is effective in inhibiting the pathway activation and growth of these cell lines under both anchorage-dependent and anchorage-independent culture conditions. Moreover, in those cell lines in which significant growth inhibition could not be achieved with a single kinase inhibitor treatment (H1993, H358, and H1650), a combination treatment with two kinase inhibitors, one targeting the RTK and the other targeting a downstream kinase, was effective in blocking their proliferation. However, we noted that there was no direct correlation between the IC_50_ value of the drug with the level of the corresponding pathway activation because we only measured a subset of pathway biomarkers. There might be other important pathways which may contribute to tumor growth. Another aspect is that the mutational status of tumor cells also plays an important role in driving the tumor growth. Nevertheless, characterization of the activated signaling pathways and mutational status as well as their cell growth inhibition by kinase inhibitors in the lung tumor cell lines could facilitate the target-focused treatment of lung cancers. 

Pathway profiling using the CEER assay and gene mutational analysis of the 50 tumor tissues collected from lung cancer patients clearly showed some similarities in the biomarkers between the lung tumor tissue samples and tumor cell lines. Therefore, the panel of pathway biomarkers and mutated genes can discriminate and cluster different tumor tissue samples with the corresponding tumor cell lines in both supervised and unsupervised clustering analysis and this information could be used to guide treatment options based on the drug sensitivity of the tumor cell lines. However, it is important to interpret the data with caution due to the small sample size of the tumor tissue samples. Prospective validation studies with sufficient sample size and enough analytical power are essential to substantiate the role of these pathway biomarkers in cancer diagnosis and treatment. The ultimate validation of this approach is the patient's response to drug treatment based on the biomarker prediction. Irrespective of the slow progress made towards curing cancer, we have gained much knowledge through translational research by using new molecular and biological technology. We believe that the continuing gain in knowledge of lung cancer biology will provide the foundation for improvement in lung cancer treatment.

## Supplementary Material

The Supplementary Material provides a detailed description of the CEER platform methodology and the CEER data generated from testing the 8 human lung tumor cell lines and 50 human lung tumor samples.

## Figures and Tables

**Table 1:** Clinical features of the eight lung tumor cell lines.

Name	ATCC Cat. no.	Type	Source
HCC827	CRL-2868	Adenocarcinoma	Primary, lung epithelial
H1975	CRL-5908	Adenocarcinoma	Primary, lung epithelial
H1734	CRL-5891	Adenocarcinoma	Primary, lung epithelial
H1993	CRL-5909	Adenocarcinoma	Metastatic, lymph node
H358	CRL-5807	Bronchioalveolar carcinoma	Primary, lung epithelial
H1650	CRL-5883	Bronchioalveolar carcinoma	Metastatic, pleural effusion
A549	CCL-185	Adenocarcinoma	Primary, lung epithelial
H460	HTB-177	Large-cell carcinoma	Metastatic, pleural effusion

## Abbreviations

SCLC: Small cell lung cancer
NSCLC: Nonsmall cell lung cancer
TKI: Tyrosine kinase inhibitor
RTK: Receptor tyrosine kinase
CEER: Channel enzyme enhanced reaction
FBS: Fetal bovine serum
PBS: Phosphate buffered saline
BSA: Bovine serum albumin.

## References

[1] Jemal A, Bray F, Center MM, Ferlay J, Ward E, Forman D (2011). Global cancer statistics. *CA Cancer Journal for Clinicians*.

[2] Vaporciyan AA, Nesbitt JC, Lee JS (2000). *Holland-Frei Cancer Medicine*.

[3] Roggli VL, Vollmer RT, Greenberg SD, McGavran MH, Spjut HJ, Yesner R (1985). Lung cancer heterogeneity: a blinded and randomized study of 100 consecutive cases. *Human Pathology*.

[4] Schiller JH, Harrington D, Belani CP (2002). Comparison of four chemotherapy regimens for advanced non-small-cell lung cancer. *The New England Journal of Medicine*.

[5] Comis RL (2005). The current situation: erlotinib (Tarceva) and gefitinib (Iressa) in non-small cell lung cancer. *The Oncologist*.

[6] Ramalingam S, Sandler AB (2006). Salvage therapy for advanced non-small cell lung cancer: factors influencing treatment selection. *The Oncologist*.

[7] Papaetis GS, Syrigos KN (2009). Sunitinib: a multitargeted receptor tyrosine kinase inhibitor in the era of molecular cancer therapies. *BioDrugs*.

[8] Neal JW, Sequist LV (2010). Exciting new targets in lung cancer therapy: ALK, IGF-1R, HDAC, and Hh. *Current Treatment Options in Oncology*.

[9] Serra V, Scaltriti M, Prudkin L (2011). PI3K inhibition results in enhanced HER signaling and acquired ERK dependency in HER2-overexpressing breast cancer. *Oncogene*.

[10] Gong HC, Honjo Y, Nangia-Makker P (1999). The NH2 terminus of galectin-3 governs cellular compartmentalization and functions in cancer cells. *Cancer Research*.

[11] Pao W, Miller VA, Politi KA (2005). Acquired resistance of lung adenocarcinomas to gefitinib or erlotinib is associated with a second mutation in the HER1 kinase domain. *PLoS Medicine*.

[12] Le Calvez F, Mukeria A, Hunt JD (2005). TP53 and KRAS mutation load and types in lung cancers in relation to tobacco smoke: distinct patterns in never, former, and current smokers. *Cancer Research*.

[13] Pao W, Wang TY, Riely GJ (2005). KRAS mutations and primary resistance of lung adenocarcinomas to gefitinib or erlotinib. *PLoS Medicine*.

[14] Sjöblom T, Jones S, Wood LD (2006). The consensus coding sequences of human breast and colorectal cancers. *Science*.

[15] Potti A, Dressman HK, Bild A (2006). Genomic signatures to guide the use of chemotherapeutics. *Nature Medicine*.

[16] Pao W, Miller V, Zakowski M (2004). EGF receptor gene mutations are common in lung cancers from “never smokers” and are associated with sensitivity of tumors to gefitinib and erlotinib. *Proceedings of the National Academy of Sciences of the United States of America*.

[17] Lynch TJ, Bell DW, Sordella R (2004). Activating mutations in the epidermal growth factor receptor underlying responsiveness of non-small-cell lung cancer to gefitinib. *The New England Journal of Medicine*.

[18] Kwak EL, Sordella R, Bell DW (2005). Irreversible inhibitors of the EGF receptor may circumvent acquired resistance to gefitinib. *Proceedings of the National Academy of Sciences of the United States of America*.

[19] Paez JG, Jänne PA, Lee JC (2004). HER1 mutations in lung cancer: correlation with clinical response to gefitinib therapy. *Science*.

[20] Kobayashi S, Boggon TJ, Dayaram T (2005). HER1 mutation and resistance of non-small-cell lung cancer to gefitinib. *The New England Journal of Medicine*.

[21] Sebolt-Leopold JS (2008). Advances in the development of cancer therapeutics directed against the RAS-mitogen-activated protein kinase pathway. *Clinical Cancer Research*.

[22] Rusnak DW, Alligood KJ, Mullin RJ (2007). Assessment of epidermal growth factor receptor (HER1, ErbB1) and HER2 (ErbB2) protein expression levels and response to lapatinib (Tykerb, GW572016) in an expanded panel of human normal and tumour cell lines. *Cell Proliferation*.

[23] Paull KD, Shoemaker RH, Hodes L (1989). Display and analysis of patterns of differential activity of drugs against human tumor cell lines: development of mean graph and COMPARE algorithm. *Journal of the National Cancer Institute*.

[24] McDermott U, Sharma SV, Dowell L (2007). Identification of genotype-correlated sensitivity to selective kinase inhibitors by using high-throughput tumor cell line profiling. *Proceedings of the National Academy of Sciences of the United States of America*.

[25] Nakatsu N, Nakamura T, Yamazaki K (2007). Evaluation of action mechanisms of toxic chemicals using JFCR39, a panel of human cancer cell lines. *Molecular Pharmacology*.

[26] Singh S, Liu X, Lee T (2010). Analysis of truncated HER2 expression and activation in breast cancer. *Journal of Clinical Oncology, ASCO Annual Meeting*.

