# Supplementary material for: Signatures of Drug Sensitivity in Nonsmall Cell Lung Cancer
Source: Int J Proteomics. 2011 Aug 7;2011:215496. doi: 10.1155/2011/215496 (PMC3200133; doi:10.1155/2011/215496)
Supplement: Supplementary file 1 — The Supplementary Material provides a detailed description of the CEER platform methodology and the CEER data generated from testing the 8 human lung tumor cell lines and 50 human lung tumor samples. [file 215496.f1.pdf]

SUPPLEMENTAL DATA AND METHODS

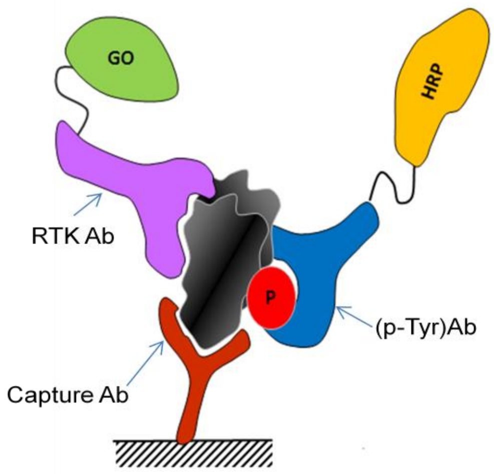

Supplemental Figure 1. Illustration of the CEER™ assay principle. The assay is based on the formation of an immuno-complex that requires the co-localization of two detecting antibodies against the captured target protein shown in black.

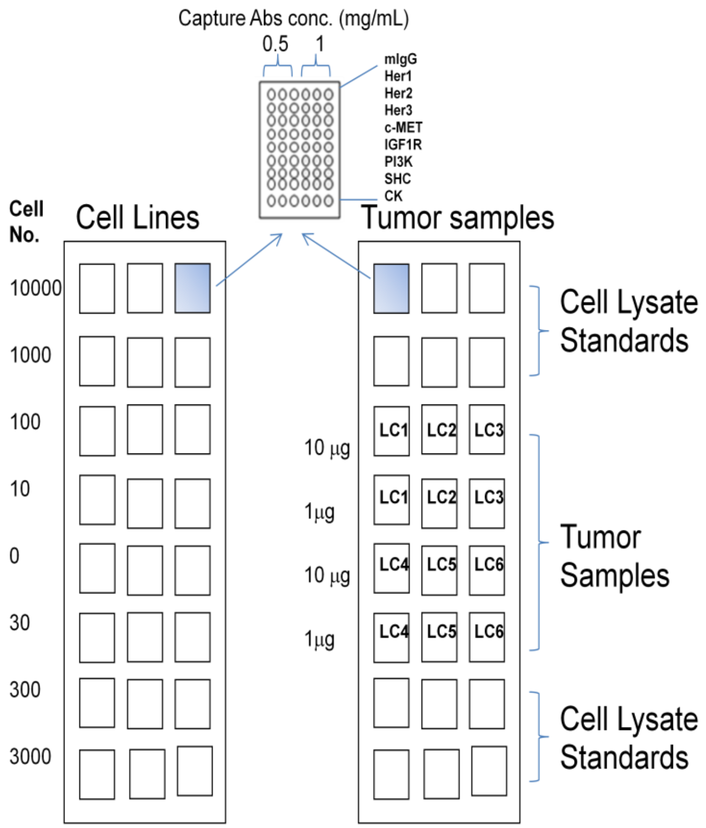

Supplemental Figure 2. Illustration of the CEER^{TM} assay protocol for determination of the activated signaling pathways in lung tumor cell lines (left panel) and lung tumor tissue samples and cell lines (right panel).

Supplemental Table 1. Clinical Characteristics of 50 human lung tumors

**Average Age of Patients (range)**	68.2 (51-90)
**Gender**	
Female	24
Male	26
**Tumor Stage**	
I	32
II	7
III-IV	11
**Current Status of Patients**	
Dead	23
Alive	27

Supplemental Table 2. Relative activation levels of the RTK pathways determined by the CEER^{TM} assay in the eight lung tumor cell lines expressed as EC_{50} values based on the number of cells' lysate being assayed.

Cell line	HER1	HER2	HER3	c-MET	IGF-1R	PI3K	SHC
HCC827	21	353	19,203	108	6,961	6,254	
H1975		1,390		1,760			4670
H1734	3,996						
H1993		167	31,202	<10			408
H358	4,383	5,108		547074	10,3267		
H1650	3,214	1,705					
A549	1,234	6,169		2,950	1,730		
H460					206,648		

Supplemental Table 3. Profiling of signaling pathways by the CEER^{TM} assay and mutational status in lung tumor samples and lung tumor cell lines. The numbers in the table correspond to the computed cell units (CU) determined for each sample and the higher the number the more activated the pathway. The mutation status of the selected genes refers to nucleotide changes in the DNA (WT=wild type, Mut= mutated and N/A= status cannot be determined)

Samples	HER1	HER2	HER3	c-MET	IGF1R	PI3K	SHC	**KRAS** (G34A, G37T)	**KRAS** (A183T)	**P53** (C726G, G818T)	**STK11** (C109T, G595T)
LC1	15.8	1.5	46.5	1.5	12.1	10.8	9.5	WT	WT	WT	WT
LC2	20.6	1.3	102.9	0.9	12.1	29.5	8.9	WT	WT	WT	WT
LC3	1.9	0.3	10.1	0.2	0	0	1.5	WT	WT	Mut	WT

LC4	2.7	0.6	9	0	1.7	1.2	7.4	Mut	WT	WT	WT
LC5	0	1.7	1.6	0.5	2.6	5.5	1.5	WT	WT	WT	WT
LC6	13.9	2.2	28.8	2.6	11.7	4.6	13.8	WT	WT	Mut	WT
LC7	0	1.9	5	0.9	5.1	11.6	2	Mut	WT	WT	WT
LC8	23.7	1.1	39.5	2.6	20.5	20.9	17.1	WT	WT	WT	WT
LC9	0.7	0.4	7.1	0.4	1	1	6.3	WT	WT	WT	WT
LC10	14.9	1.4	58.2	0.3	1.3	0.8	2.2	WT	WT	Mut	WT
LC11	16.8	4.3	39.4	1.2	10.7	11.2	10.5	WT	WT	N/A	WT
LC12	43.6	1.1	67.5	0.6	18.2	11.4	22.9	WT	WT	WT	WT
LC13	20.6	0.7	42.7	0.5	14.3	20.4	6.9	WT	WT	Mut	WT
LC14	17.6	1.3	46.1	2.6	36.6	21.3	21.2	WT	WT	WT	WT
LC15	3.1	0.2	4	0.2	0.6	3.4	1.1	WT	Mut	WT	WT
LC16	18.2	0.8	38	1.5	26.8	18.6	3.9	Mut	WT	WT	WT
LC17	3.6	0.2	827	0.1	7.5	1.3	2.9	WT	WT	N/A	WT
LC18	9	1.1	11.8	0.8	10.2	18.5	6.2	WT	WT	WT	WT
LC19	7.5	2.2	324.8	2	4.9	22.6	52.3	WT	WT	WT	WT
LC20	9.5	1.4	41.1	0.3	2.1	0.8	1.7	Mut	WT	WT	WT
LC21	0	2.4	4.9	1.1	2.9	6.3	7.5	Mut	WT	WT	WT
LC22	0	0	0	0.6	0	0	0	WT	WT	WT	WT
LC23	22.6	0.4	251.3	0.1	5.8	24.9	4.9	Mut	N/A	Mut	WT
LC24	10.8	0.5	35.1	0.4	11	4.9	0.2	WT	WT	N/A	WT
LC25	26.7	2.9	47.6	2.3	19.1	33.3	28.3	WT	N/A	WT	Mut
LC26	13	1.3	31.6	1.1	16.2	26.6	15.7	WT	N/A	WT	WT
LC27	14.3	0.7	46.7	0.1	6.2	3.4	8.7	WT	WT	WT	WT
LC28	8.1	1.8	501.6	0.1	2.7	11.2	175.3	WT	N/A	WT	WT
LC29	16.4	0.2	535.7	0.4	0.5	0.4	1.3	WT	WT	Mut	WT
LC30	4.1	0.2	5.1	0	1.3	1.2	0	Mut	WT	WT	WT
LC31	70	5.1	99.7	7.8	36.2	69.5	75.4	WT	N/A	Mut	WT
LC32	16.5	0.9	29.5	1.3	16.9	15.4	5.2	N/A	WT	Mut	WT
LC33	19.3	0.6	78.3	0.1	8.6	1.6	10.4	WT	WT	WT	WT
LC34	16.1	0.6	21.4	1.6	10.1	17.8	5.8	WT	WT	WT	WT
LC35	0	0.8	29.4	0.1	0	0	5.6	WT	WT	WT	WT
LC36	18.9	0.9	61.6	0.3	7.2	2.9	8.9	WT	WT	Mut	WT
LC37	9.3	0.8	25.9	0.7	5	0	11	WT	WT	WT	WT
LC38	0	1.6	12	1.3	8.1	16.1	3.4	WT	WT	WT	WT
LC39	11.8	2.7	31.1	0.7	9.9	14.8	3	WT	WT	WT	WT
LC40	27.7	2.4	49.8	2.1	16.1	12.3	25.4	N/A	N/A	N/A	WT
LC41	0	1.5	4.2	0.9	2.9	6.6	1.6	WT	WT	WT	WT
LC42	13.5	1.3	49	0	10	38.3	4.1	WT	N/A	WT	WT

LC43	19.3	2.1	26.4	1.4	13.5	23.7	10.7	WT	N/A	WT	WT
LC44	10.3	0.5	28.1	1.1	13.3	9.2	2.5	Mut	WT	WT	WT
LC45	128.7	13.4	239.1	3.2	20.4	81.4	96.2	Mut	WT	WT	WT
LC46	4.3	0.4	13.6	0.1	2.2	2.4	1.2	WT	WT	WT	WT
LC47	26.5	2.6	83.6	0.9	31.6	68.4	21.2	WT	WT	WT	WT
LC48	0	0.1	4.2	0.1	2.4	3.3	4.8	WT	WT	WT	WT
LC49	15.8	2.6	15.9	0.2	1.7	1.2	5.6	WT	WT	WT	WT
LC50	16.8	0.7	28.7	1.2	9	27.8	15.7	WT	WT	WT	WT
H1993	24.4	7.3	97.5	11.2	2.2	60.9	31.6	WT	WT	Mut	Mut
H1975	15.6	5.5	329.8	2.1	9.2	53.8	44.9	WT	WT	Mut	WT
HCC827	20.3	6.6	530.9	2.5	10.5	48.6	31.7	WT	WT	WT	WT
H1734	11.2	2.8	125.9	1.7	5	18.4	11	Mut	WT	Mut	WT
H1650	12.2	1.4	21.9	0.2	2.5	10.8	13.7	WT	WT	Mut	WT
H460	0.2	0.1	3.1	0.7	1.1	0.4	1.9	WT	Mut	WT	Mut
H358	4.4	0.9	14.8	1.5	9.2	15.5	3	Mut	WT	WT	WT
A549	15.8	0.6	22	1.6	11.5	16.3	4.9	Mut	WT	WT	Mut

Supplemental CEER^{TM} assay procedure for profiling of signaling pathways in tumor cell lines:

The assay was performed by printing 500 pL of a commercially available capture antibody per spot at two dilution concentrations of 0.5 and 1.0 mg/mL in triplicates to yield six spots per row for each capture antibody on a 3X2 cm nitrocellulose-coated glass slide (FAST®, Whatman) using a Nano-Plotter NP2.1 printer (GeSIM mbH, Germany) as illustrated in Supplemental Figure 2, upper panel. The capture antibodies against mouse IgG, human epidermal growth factor receptor 1 (HER1), human epidermal growth factor receptor 2 (HER2), human epidermal growth factor receptor 3 (HER3), human hepatocyte growth factor receptor (c-MET), human insulin-like growth factor-1 receptor (IGF1R), human stem cell growth factor receptor (cKit), human phosphatidylinositol-3-kinase (PI3K), human Src homology 2 domain-containing protein (SHC) and human cytokeratin (CK), were provided by reputable suppliers which had provided western blotting data demonstrating that the antibodies are specific for the particular proteins. The antibodies were printed in this order in 10 rows from top to

bottom in each slide. In the assay of the tumor tissue samples, printing of the capture antibody against cKit was omitted because activation of this signaling pathway was not observed in any of the lung cancer cell lines. The anti-mouse-IgG printed on the first row was used as a negative control while the pan-cytokeratin capture antibody printed on the last row was used as a positive control. After printing, the slides were rinsed 2 times with TBST (50 mM Tris/150 mM NaCl/0.1% Tween-20, pH 7.2-7.4), blocked with 80 µL of Whatman Blocking Buffer (Whatman) for 1 hr at room temperature (RT), and then washed 2 times with TBST. For measurement of the activated pathways in the lung cell lines, serially diluted cell lysates in 80 µL of reaction buffer (2% bovine serum albumin (BSA)/0.1% Triton X-100/TBS [TBST without Tween-20], pH 7.2-7.4) were added to the slides according to the number of cells' lysate being assayed as illustrated in Supplemental Figure 2, left panel, and incubated for 1 hr at RT. After incubation, the slides were washed 4 times with TBST, 3 min each time. Then a combination of each pair of respective commercially available detecting antibodies for each of the targeted kinases, one conjugated to GO and the other to HRP, were added in 80 µL of the reaction buffer followed by incubation for 2 hrs at RT. The unbound detecting antibody conjugates were removed by washing with TBST. Then, 80 µL of the signal generation and amplification solution (5 µg/mL biotinyl tyramide [Perkin Elmer Life Sciences] in 50 mM glucose in PBS) was added to each slide and incubated for 15 min in the dark. The slides were then washed with TBST 4 times, with 3 min each time. The local deposition of the generated biotin-tyramide signal was revealed upon the addition of a signal-detecting reagent, streptavidin-labeled Alexa647 (Invitrogen) in PBS at 0.5 µg/mL (1:4000 dilution) in 2% BSA/0.1% Triton/TBS and incubated for 40 min. Upon completion of the incubation, the slides were washed 4 times with TBST, dried, and kept in the dark until scanning on the microarray scanner. Each slide was scanned at three photomultiplier gain settings to increase the effective dynamic range. Background corrected signal intensities were averaged for replicate spots printed in triplicate. The relative fluorescence value of the respective reagent blank was subtracted from each sample to give a relative fluorescence unit (RFU) according the number of cells' extract. The calculated RFUs with the corresponding number of cells' extracts were used to generate the dose-response pathway activation curves for each cell line.

Supplemental CEER™ assay procedure for profiling of signaling pathways in tumor tissue samples:

For comparison of the relative levels of signaling pathway activation within the 50 tumor tissue samples and the eight tumor cell lines, we used a mixture of three cell lines, BT474, T47D and HCC827, as a standard to generate the dose-response curves for each of the activated pathways profiled by the CEER™ assay. BT474 is a breast cancer cell line from ATCC (Cat. No. HTB-20) exhibiting a strongly activated HER2 pathway. T47D is another breast cancer cell line from ATCC (Cat. No. HTB-133) that showed robustly activated HER3 and IGF1R pathways while, HCC827, a lung tumor cell line employed in the present study was selected because it exhibited a highly activated HER1 and c-MET pathway. To generate the standard curves, lysates corresponding to 300 BT474 cells, which had been pretreated with 10 nM Heregulin for 15 min, 1000 T47D cells, which had been pretreated with 100 ng/mL IGF-1 for 15 min, and 1000 HCC827 cells, which had been pretreated with 100 nM EGF for 15 min, were combined to give the starting standard cell lysate concentration, which was then serially diluted to yield a total of six concentrations based on the original number of cells employed to produce the lysate. Each of the original and diluted cell lysate concentrations, according to decreasing cell numbers, was added to the six capture antibody pre-printed slides on the top and bottom two rows in the panel with one lysate concentration per slide as illustrated in Supplemental Figure 2, right panel. By combining these three cell lysates, we generated signals to cover most of the activated pathways detected in the tumor cell lines. Each subsequent two rows of slides in the middle of the panel were loaded with extracts from the tumor tissue samples or tumor cell lines with the top row corresponding to 10 µg and the bottom row corresponding to 1 µg protein concentration and one sample per column. After loading of the slides in each panel, they were subjected to the CEER™ assay as described before and the generated data from the lysate standards were fitted to a five parameter equation by nonlinear regression, simultaneously fitting two dilutions of the capture antibody, to generate the standard

curves for each pathway. The individual computed cell unit (CU) for the tumor tissue samples or cell lines in each pathway was determined from the standard curves.

Supplemental method for preparation of the antibody/GO conjugate and the anti-p-Tyr/HRP conjugate:

The antibody/enzyme conjugate was linked through a dextran molecule incorporated with free sulfhydryl groups. The sulfhydryl-incorporated dextran was prepared by first converting some of the hydroxyl groups on a 500 KDa dextran molecule to carboxymethyl ether groups through reaction with bromoacetic acid in a sodium hydroxide solution. The incorporated carboxymethyl grouping was then coupled to the amino functional group of cysteamine ($HSCH_2CH_2NH_2$) through water soluble carbodiimide to yield the sulfhydryl-incorporated dextran. To conjugate the respective antibody and enzyme to the sulfhydryl-incorporated dextran, the antibody or enzyme was first activated through coupling of the free amino functional groups on the antibody or enzyme with a bi-functional cross-linker, succinimidyl-4-(N-maleimidomethyl)-cyclohexane-1-carboxylate (SMCC, Pierce) according to the manufacturer's instruction. Using a 1:5 molar ratio of antibody to enzyme, the resulting maleimidomethyl incorporated antibody and enzyme are then reacted simultaneously with the sulfhydryl incorporated dextran to form the antibody-enzyme conjugate, which was then purified by size-exclusion high-performance liquid chromatography.
